# Two-photon excitation fluorescence microspectroscopy protocols for examining fluorophores in fossil plants

**DOI:** 10.1038/s42003-024-05763-z

**Published:** 2024-01-06

**Authors:** Michael R. Stoneman, Victoria E. McCoy, Carole T. Gee, Katherine M. M. Bober, Valerică Raicu

**Affiliations:** 1 Department of Physics, University of Wisconsin-Milwaukee, Milwaukee, WI 53211 USA; 2 Department of Geosciences, University of Wisconsin-Milwaukee, Milwaukee, WI 53211 USA; 3 School of Geography, Geology, and the Environment, University of Leicester, Leicester, LE1 7RH UK; 4 Institute of Geosciences, Division of Paleontology, University of Bonn, Nussallee 8, 53115 Bonn, Germany

**Keywords:** Palaeontology, Palaeoecology

## Abstract

Fluorescence emission is common in plants. While fluorescence microscopy has been widely used to study living plants, its application in quantifying the fluorescence of fossil plants has been limited. Fossil plant fluorescence, from original fluorophores or formed during fossilization, can offer valuable insights into fluorescence in ancient plants and fossilization processes. In this work, we utilize two-photon fluorescence microspectroscopy to spatially and spectrally resolve the fluorescence emitted by amber-embedded plants, leaf compressions, and silicified wood. The advanced micro-spectroscope utilized, with its pixel-level spectral resolution and line-scan excitation capabilities, allows us to collect comprehensive excitation and emission spectra with high sensitivity and minimal laser damage to the specimens. By applying linear spectral unmixing to the spectrally resolved fluorescence images, we can differentiate between (a) the matrix and (b) the materials that comprise the fossil. Our analysis suggests that the latter correspond to durable tissues such as lignin and cellulose. Additionally, we observe potential signals from chlorophyll derivatives/tannins, although minerals may have contributed to this. This research opens doors to exploring ancient ecosystems and understanding the ecological roles of fluorescence in plants throughout time. Furthermore, the protocols developed herein can also be applied to analyze non-plant fossils and biological specimens.

## Abstract (web summary)

New protocols allow the collection of fluorescence excitation and emission spectra from a variety of fluorescent plant megafossils, enabling research into the underlying fluorescent molecules, including potential remnants of original fluorescence.

## Introduction

Fluorescence is widespread among extant plants, primarily due to the commonality of aromatic rings within organic matter, which act as fluorophores that absorb and emit light across the visible spectrum^1–3^. It is also believed that fluorescence in plants is also used as a visual signal to communicate with animals^4,5^. Although the importance of fluorescence in visual signaling is still under debate^2,5,6^, a number of possible roles have been suggested and are supported by empirical or experimental studies^1,7^: fluorescence signaling by flowers or pollen to attract pollinators; fluorescence signaling by ripe fruit or seeds to attract seed-dispersing mammals; fluorescence signaling by carnivorous plants to attract arthropod prey; and fluorescence signaling in senescent leaves to warn insects of chemical defenses and prevent egg-laying. Plants have also developed a number of features to protect their photosynthetic apparatus from damaging UV radiation, such as secondary metabolites that strongly absorb UV radiation^1^. These fluorophores do not just absorb UV light but also re-emit light at wavelengths accessible to photosynthesis^1^. Therefore, fluorophores may also serve a dual purpose of protecting from UV radiation and enhancing photosynthesis^1^.

Fluorescence was presumably similarly widespread and important among plants in ancient ecosystems, but it remains understudied. Many fossil plants do fluoresce, and biomolecules are known to be preserved in fossils^8^, suggesting that the fluorescence observed in fossil plants could be the result of the preservation of original fluorophores. However, there is currently very little information in the literature about fluorescence emission (i.e., fluorescence intensities vs. emission wavelength, or color) and excitation spectra of the fluorophores preserved in fossil plants, as fluorescence in plant megafossils has only rarely been studied quantitatively or interpreted to identify the molecular content of the fossils^9^. In spite of this scarcity of data, there is already some evidence that fossil plant fluorescence can be interpreted in a biological context. For example, Wolkenstein and Arp^9^ reported widespread fluorescence of plant compressions from the upper Pliocene Willershausen fossil site. Different taxa emitted different colors, and these were attributed to broad fluorophore groupings; variations in the fluorescence were largely due to the breakdown of organic compounds during leaf senescence, a process that varies taxonomically^9^.

The fluorescence of fossil plants could also be caused by fluorophores produced through chemical changes during fossilization. For example, amber, or fossilized tree resin^10^, commonly fluoresces; however, the fluorescence in amber is thought to be produced by fluorescent aromatic hydrocarbons that develop during the fossilization process by which resin matures over millions of years into amber^11^. Most commonly, studies of the fluorescence in fossil plant material focus on changes that occur during fossilization and are used to infer details of diagenetic alteration; for example, fluorescence can reveal the thermal maturity of fossil pollen samples^9^.

Regardless of whether fluorophores are original or produced during fossilization, fluorescence spectroscopy offers an exquisite means to identify the material^1–3,7,12^. Several factors contribute to the overall fluorescence emission spectrum of a particular material—including the presence of mixtures of fluorophores with varying relative abundances, as well as the optical properties of the tissue or material—and tissues or biological and non-biological structures often exhibit characteristic fluorescence^1,3,13^. Quantification of the fluorescence emission spectra of fluorescent fossil plants, therefore, could also provide valuable information about the composition of the fossils. For example, laser-induced fluorescence spectroscopy, in combination with Raman spectroscopy, of leaf compressions from a range of fossil sites, revealed that the fossils had undergone chemical changes during fossilization and in particular that these fossils contain oxidized forms of chlorophyll derivatives^14^. The scarcity of previous fluorescence spectroscopy studies on fossil plants^9^ can be largely attributed to the lack of well-developed methods. For example, Bunkin et al. noted that they used laser-induced fluorescence spectroscopy in their study because they were unable to gather usable data from conventional fluorescence spectroscopic methods^14^.

The goal of our study here is to develop protocols for applying two-photon fluorescence microspectroscopy^15,16^ to fossil plant organs that are preserved in various ways to characterize, quantify, and interpret their fluorescence. Botanical fossils are the endpoint of widely separate fossilization pathways^17^. As a result, each pathway may vary in how effectively it preserves fluorophores^18^, and each type of fossil may represent unique methodological challenges. Herein, we focus specifically on amber and bio-inclusions in amber, fossil leaf compressions, and silicified wood.

A combination of three features in the microspectroscope used in the present study are essential for accurately characterizing the excitation and emission spectra of plant fossils: (i) pixel-level spectral resolution, (ii) two-photon excitation, and (iii) line-scan excitation. Pixel-level spectral resolution enables the distinction of molecular species possessing different emission spectrum profiles, even near the boundaries between adjacent sample features. When combined with spectral deconvolution (or unmixing), it enables the quantification of different types of fluorescence sources, even if they reside in the same image pixel. Two-photon excitation employing pulsed near-infrared light enables deep penetration of samples along with image-sectioning capability, i.e., intervening layers do not contribute to the signal originating from the depth at which the fluorophores of interest reside. A secondary feature of two-photon excitation is the substantial spectral separation (>350 nm) between the laser’s excitation wavelength and the emission spectrum of the fluorescing species, regardless of the specific species under study. This large spectral separation simplifies the optical setup, allowing for a consistent spectral range to be probed across various excitation wavelengths, valuable for accommodating different fluorophores and incorporating excitation spectra into the measurements. Line-scan excitation allows for the simultaneous excitation of hundreds of voxels, similar to operating hundreds of individual imaging instruments concurrently. As a result, when the total time for acquiring an entire field of view is kept constant between the line-scan and traditional point-scanning methods, the line-scan approach collects signal levels orders of magnitude higher due to a much longer pixel dwell time. This increase in signal level is attributed to the longer relative exposure time for each individual pixel. The notable boost in signal levels facilitates a considerable reduction in excitation power, a critical factor when imaging fossil plant specimens susceptible to photobleaching as well as to laser damage. Therefore, to mitigate the risk of photobleaching and photodamage in this study, we employ the line-scan approach, which allows us to maximize signal collection while reducing the power-per-voxel and the acquisition time as much as possible. These protocols therefore provide a nondestructive and spatially resolved way to chemically analyze fluorescent molecules in fossil plants. They will provide a valuable complement to typical destructive and less-spatially resolved methods of analysis, such as gas chromatography-mass spectrometry for characterizing the organic composition of fossils.

## Results

### Fossil leaf compressions

Leaf compression fossils are very common and vary widely in preservational quality. However, some of the best-preserved leaf compressions, for example, those from the Miocene Clarkia fossil deposit in Idaho, exhibit the exceptional preservation of ancient biomolecules and organic biomarkers^19–21^. The exceptional chemical preservation of the dicot leaves at this site suggests that fluorophores may also be similarly well-preserved. In particular, some of the chemical compounds previously identified in these ancient leaves, such as chlorophyll derivatives and flavonoids, do fluoresce in living plants^3,22^. Some Clarkia leaf compressions have been observed to fluoresce under epifluorescence microscopy^9,23^ although the fluorescence emission and excitation spectra have not been quantified.

To effectively detect and characterize the fluorescence of leaf compressions, we utilized a previously described two-photon optical microspectroscopy technique^15,16,24^. This technique involves scanning a focused laser beam across a fossil sample and capturing fluorescence spectra at the pixel level for each scanned location. However, it is important to note that scanning a leaf compression fossil with light beams focused into single points carries a high risk of damaging the specimen. Such potential damage can lead to deformations or complete destruction of the fossil leaf, rendering it unsuitable for further study or analysis.

To minimize the risk of photodamage, we implemented a configuration of our two-photon optical microspectroscope that utilizes a line-scan excitation of the sample^15^. Unlike traditional scanning systems, which excite one voxel at a time (e.g., in confocal microscopes), the line-scan approach simultaneously excites all voxels along an entire line, resulting in a remarkable increase in the scanning and photon collection speed by multiple orders of magnitude. This substanstial improvement in photon collection efficiency allows for a notable reduction in the amount of power-per-pixel required to excite the sample, thereby minimizing the potential damage to the samples. Potential photodamage of the leaf compressions was assessed by imaging a small spot in a sample multiple times and checking for major alterations in sample morphology. Following this test, we chose an average excitation power of 0.2 mW per pixel and an exposure time of 35 ms per pixel, which allowed us to perform microspectroscopic scans of 1-mm^2^ areas on the leaf compression within 27 seconds.

The presence of a wide spectral detection window proves to be particularly valuable when examining the leaf compressions, which are typically embedded in and surrounded by a matrix consisting of various minerals and organic matter. Each mineral or organic component has the potential to contribute fluorescence to the detected signal, resulting in a high “background” signal. By leveraging the broad spectral detection window and high spectral resolution inherent to our microspectroscope, and by employing a previously described spectral unmixing technique^25,26^ (given in greater detail in the Materials and Methods section) we effectively separated the fluorescence signal of interest from those of the matrix components.

**Fig. 1:**
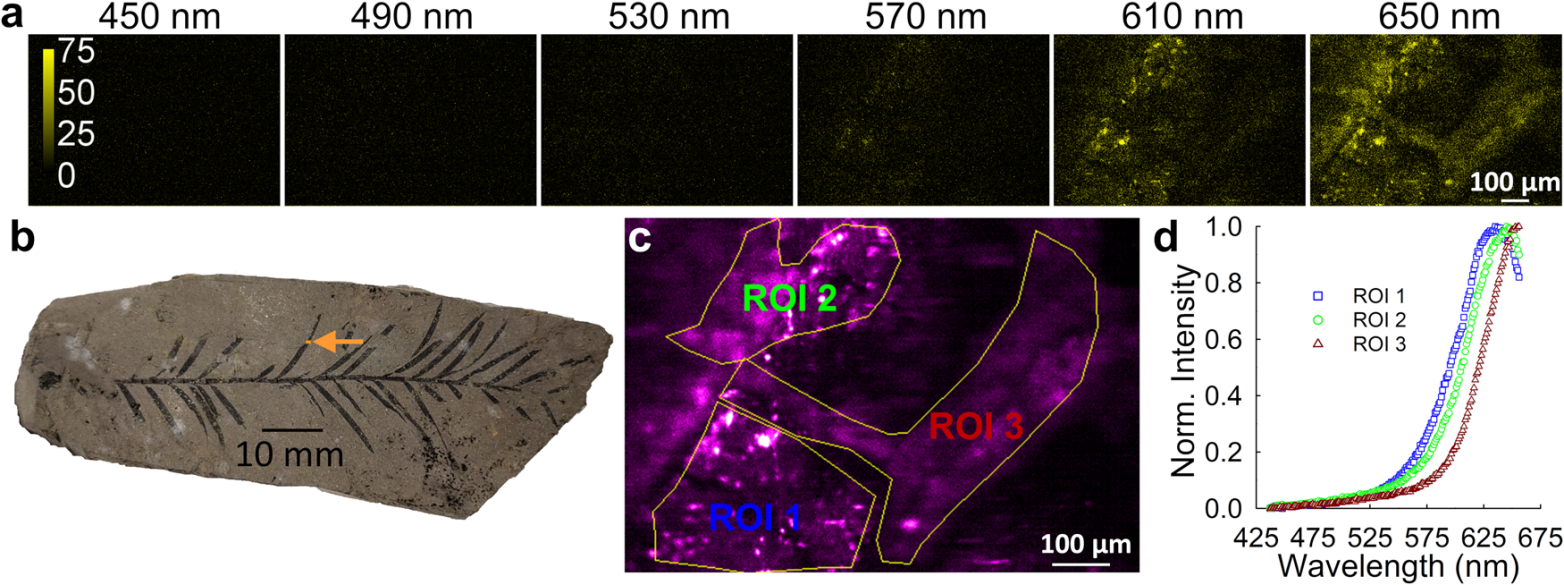
Microspectroscopic analysis of MPM Pb2853, a fossil plant compression of a *Taxodium* twig with leaves from the Miocene Clarkia fossil deposit in Idaho, USA, using two-photon excitation at 760 nm. **a** Fluorescence images captured for six select emission wavelength bands (out of a total of 200 acquired), each with a 1 nm width; the corresponding center wavelength for each band is indicated above the panel. **b** Photograph of the entire specimen. The orange arrow superimposed on the image points to the region of a leaf that was scanned to produce the images shown in A. **c** Regions of interest (yellow lines) drawn on a spatial distribution map generated by averaging the fluorescence signals obtained over 51 wavelength channels ranging from 600 nm to 650 nm for enhanced visualization only. **d** Average emission spectra corresponding to the three different regions of interest (ROI) shown in panel **c**. Each spectrum was obtained by averaging the pixel intensities within the ROI for each of the 200 wavelength channels of the microspectroscopic data set. Each spectrum may represent a mixture with different proportions of the fluorescence from organic fluorophores within the leaf (either preserved original fluorophores or fluorophores produced during fossilization), as well as from minerals or organic material in the sediment matrix.

Examples of fluorescence spectra obtained from scanning the leaf of a Miocene twig of *Taxodium* using the two-photon optical microspectroscope at an excitation wavelength of 760 nm are presented in Fig. 1. For the particular field of view displayed in Fig. 1, a stack of microspectroscopic images was obtained which contained 200 different wavelength channels, each with ~1 nm bandwidth; the size of the image for each emission wavelength channel was 440 × 300 pixel^2^. Figure 1a provides a condensed visual overview of the extensive microspectroscopic data set, presenting abundance maps derived from a subset of six wavelength channels chosen from the complete set of 200 channels, for the region of the leaf pictured in Fig. 1b. The emission spectra plotted in Fig. 1d are obtained by averaging the pixel-level emission spectra from all of the pixels contained within the corresponding region of interest (ROI) shown in Fig. 1c. The drawing of the ROI contour was guided by the largest contrast in fluorescence intensities and the requirement to maximize spectral differences between regions. There exists a noticeable variation among fluorescence spectra from the different ROIs, both in the peak wavelength and width of the individual spectrum. This indicates that a number of different fluorescing entities are present in the sample at those particular locations.

We consistently identified a spectral signature with a peak at 630 nm within the leaf compression fossil. In addition to this primary signature, a distinct spectral feature emerged from our analysis, characterized by a sharp peak appearing at around 640 nm in the emission spectrum. Interestingly, this unique emission pattern was exclusively observed in areas of the rock matrix devoid of any leaf material. From this observation, we can reasonably infer that this particular spectral component originates from materials such as minerals or organic substances present in the matrix surrounding the fossil. A previous study using remote laser-induced fluorescence to investigate various soils and rocks up to ~100,000 years old found that fluorescence in rocks such as the matrix around a fossil could be largely attributed to residual organic material^27^. It is also known that minerals can also fluoresce, especially if they contain impurities in their crystal structure, which could be an additional source of fluorescence from the fossil matrix^28,29^. Figure 1 illustrates that the emission spectra within certain regions of the leaf exhibit a partial overlap with the spectrum observed in the matrix region. The high spatial resolution accompanying the spectral resolution in our study allows us to distinguish between the different contributions. Fluorescence emission peaks within the 600–750 nm range in both modern and fossil plants are typically associated with chlorophyll or its derivatives^3,9,14^. In the present study, it may represent the intact chlorophyll-protein complex from the photosynthesis apparatus of the plant, chlorophyll itself, or chlorophyll derivatives produced during decay and fossilization^3,14^. This assertion is also supported by the fact that chlorophyll derivatives, which vary depending on how degraded the specimen is, have been identified in leaves from the Clarkia fossil site^22^. In addition, tannins are sometimes found in leaves and can fluoresce with a peak emission between 500–650 nm^3^. Like chlorophyll derivatives, tannins have also been identified in fossil leaves from Clarkia^22^.

The similarity in fluorescence emission spectra between the leaf compression fossil and its surrounding matrix observed here parallels results from a recent study using laser-induced fluorescence spectroscopy to quantify the fluorescence of leaf compressions and their surrounding matrix^14^, and it may be due to the similarity in the composition of leaf compressions and organic material in the sedimentary matrix at the Clarkia fossil site. This is supported by a previous study using gas chromatography–mass spectrometry (GC-MS) to analyze lipid extracts from the sediment and plant megafossils obtained from the P-37 site at the Miocene Clarkia deposit^30^. Analysis of conifer and angiosperm megafossils showed that angiosperm and conifer biomarkers were preserved in both the plant megafossils and in the surrounding matrix. However, while the matrix contained a mixture of all biomarkers, the plant fossils exhibited a clear segregation of angiosperm and conifer biomarkers, with angiosperm biomarkers exclusively present in the angiosperm leaves and conifer biomarkers confined to the conifer leaves^30^. Given the considerable overlap in emission spectra between the surrounding matrix and the leaf, distinguishing the specific contributions of materials within the matrix and a leaf’s fluorophores to the emission spectrum of a given pixel poses a formidable challenge. To overcome this challenge, we employed linear spectral unmixing which enabled us to selectively isolate the signal originating from the leaf, effectively separating it from the influence of the surrounding matrix.

Spectral unmixing is a computational technique used in fluorescence microscopy to separate the signals emitted by different materials that are simultaneously present in the same pixels^25,31,32^ of an image. It relies on knowledge of constituent spectra, called *elementary spectra*, each of which represents the pure spectral profile of an individual fluorescent species present in the sample. The composite spectrum at each image pixel can be accurately represented by a weighted sum of the elementary spectra. Importantly, the weights, or abundance values, assigned to each of the respective elementary spectra reflect the concentration of the fluorescent species in that pixel.

**Fig. 2:**
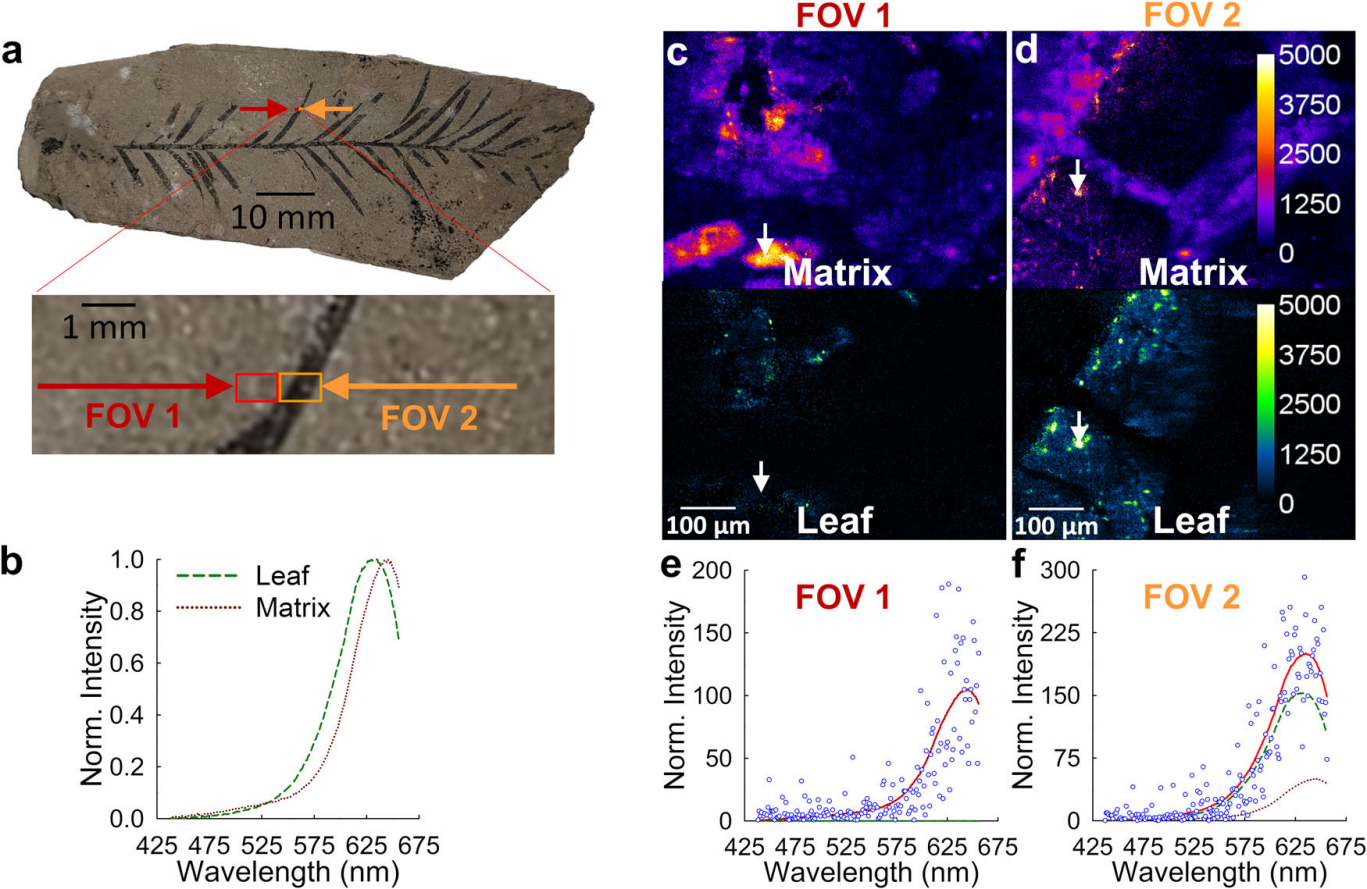
Illustration of pixel-level spectral unmixing of the microspectroscopic data set shown in Fig. 1 using two elementary spectra. (**a**) Bright-light image of MPM Pb2853, the compression of a *Taxodium* twig from the Miocene Clarkia deposit, and inset showing a magnified view of two adjacent areas investigated using two-photon excitation. The red rectangle (FOV1) indicates a field of view of the matrix for which the analysis is displayed in panels **c** and **e**. The orange rectangle (FOV2) indicates a field of view of the fossil leaf for which the analysis is displayed in panels **d** and **f**. (**b**) Plots showing the unique elementary spectra detected within FOV1 and FOV2 following two-photon excitation at 760 nm. These spectra are the purest spectral signatures that could be acquired of the individual components present in a sample region. One elementary spectrum corresponds to leaf fluorescence emission (green dashed line), while the other represents the matrix contribution (maroon dotted line). **c**, **d** Two-dimensional abundance maps obtained by applying the spectral unmixing procedure, defined by Eq. 2 in the text, to the microspectroscopic images obtained upon two-photon excitation (at 760 nm) from FOV1 (panel **c**) and FOV2 (panel **d**). **e, f** Illustration of the spectral unmixing results for a single pixel in the microspectroscopic image stacks of FOV1 (plot shown in panel **e**) and FOV2 (plot shown in panel **f**). The white arrows in each abundance map identify the selected single pixel whose spectrum was plotted. Blue circles represent measured intensities, solid red lines represent the best-fit spectra, the green dashed lines represent the leaf spectrum, and the maroon dotted lines represent the matrix spectrum.

The results of the spectral unmixing procedure applied to microspectroscopic images of a leaf compression acquired at an excitation wavelength of 760 nm, chosen for its efficiency in exciting multiple molecules which allowed us to demonstrate the unmixing of multiple fluorescent species using a single spectroscopic stack, are illustrated in Fig. 2. Detailed information on the algorithm used to perform the spectral unmixing can be found in the Materials and Methods section. Figure 2 highlights the results obtained from two adjacent fields of view (FOV) from within the specimen, labeled as FOV1 (red square in Fig. 2a) and FOV2 (orange square). Visual inspection of Fig. 2a reveals that FOV1 encompasses a region in the specimen that appears to be composed primarily of the surrounding matrix, whereas the region spanned by FOV2 is comprised of an appreciable amount of fossil leaf material along with the matrix.

For the pixel-level spectral unmixing applied to the fields of view displayed in Fig. 2, two elementary spectra were employed, each of which is plotted in Fig. 2b. One of the elementary spectra (maroon dotted line in Fig. 2b) corresponds to the unique spectral profile observed from regions of the specimen that consisted only of the surrounding matrix. The second elementary spectrum used in the spectral unmixing (green dashed line in Fig. 2b) corresponded to the fluorescence emission primarily originating from components of the fossil leaf. For each FOV, a pair of abundance maps was generated by applying the spectral unmixing procedure (defined by Eq. 2 in Materials and Methods) to the microspectroscopic images captured within the respective FOVs. These maps reflect the pixel-level abundance values of the respective materials (i.e., the matrix and leaf material) identified by each of the elementary spectra. As can be seen, the abundance maps corresponding to FOV1 (Fig. 2c) and FOV2 (Fig. 2d) reflect the qualitative assessment of each FOV given in the previous paragraph, namely that FOV1 consists primarily of matrix material while FOV2 consists of fossil leaf surrounded by matrix. Finally, displayed in Fig. 2e and Fig. 2f are examples of the spectral unmixing occurring in the single pixels indicated by the white arrows in the abundance map immediately above it.

**Fig. 3:**
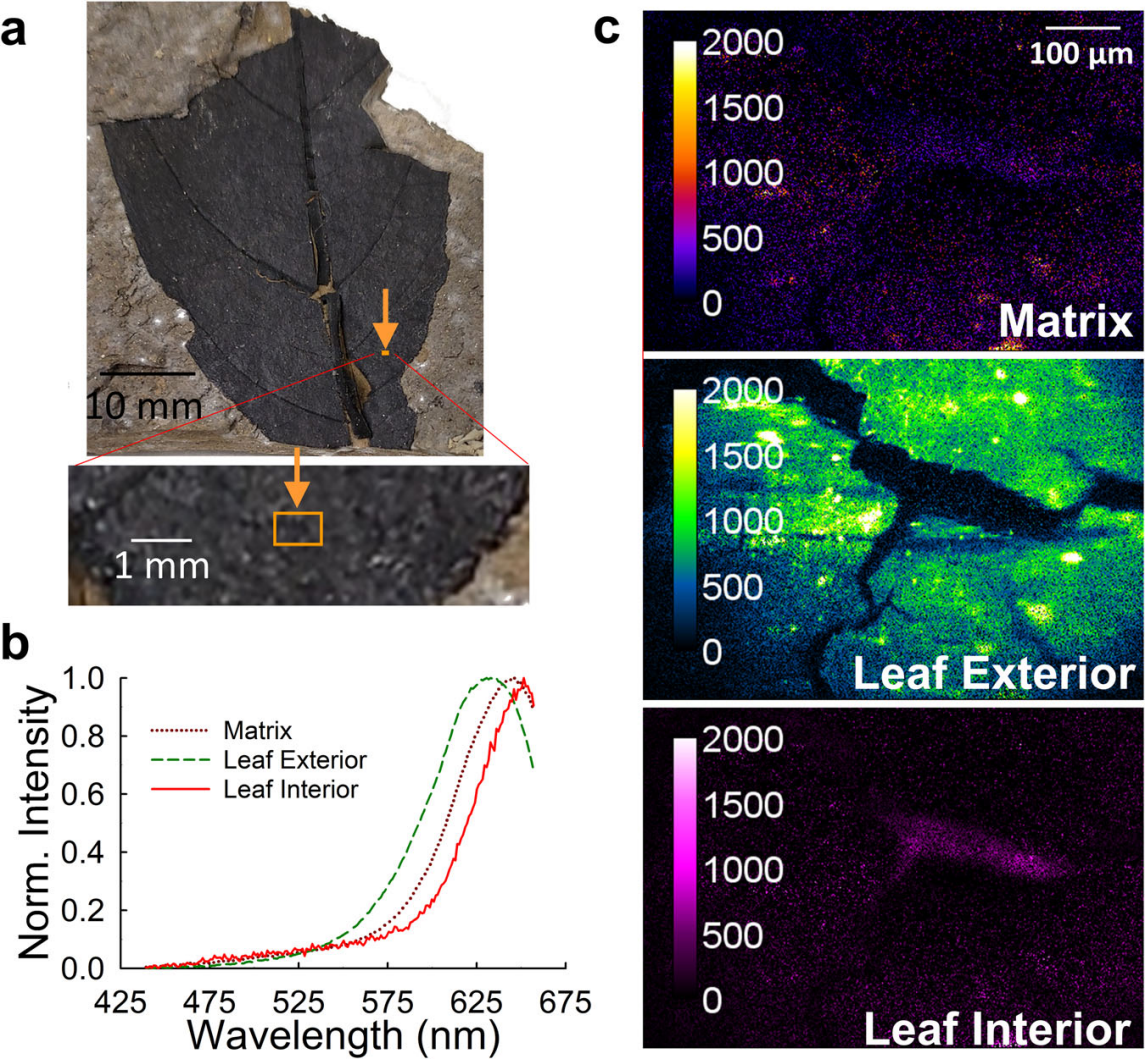
Illustration of pixel-level spectral unmixing of a microspectroscopic data set using three elementary spectra obtained for MPM Pb2854, an unidentified dicot leaf compression from the Miocene Clarkia deposit. **a** Photo of the fossil leaf and location of the laser beam (orange rectangle) for the FOV displayed in **c**. The inset provides a magnified view of the area scanned in panels **b** and **c**. (**b**) Elementary emission spectra obtained upon two-photon excitation at 760 nm. Three distinct spectral signatures have been identified: one corresponding to the matrix, one representing the outer surface of the leaf, which may include the waxy outer cuticle or epidermis of the leaf (Leaf Exterior), and one representing the internal tissue, most likely the mesophyll, located between the upper and lower epidermal layers (Leaf Interior). **c** Abundance maps obtained by applying the spectral unmixing procedure defined by Eq. 2 in the text to the microspectroscopic data set obtained upon two-photon excitation (at 760 nm) from the FOV shown in **a**. The three elementary spectra shown in b have been used for unmixing at each pixel in the spectrally resolved fluorescence micrographs. The unmixing procedure was performed using a non-negativity constraint on the abundance values (see the Linear mixture model for spectral unmixing subsection in the Materials and Methods) to ensure the accuracy of the results when using three elementary spectra with closely spaced peak wavelengths (see Supplementary Fig 1).

During the analysis of microspectroscopic stacks obtained from multiple leaf compression fossils, we encountered specific regions where spectral unmixing required the use of three elementary spectra. One such region is depicted within the orange rectangle shown in Fig. 3a, with the three elementary spectra used to unmix the spectrally resolved data obtained from this FOV plotted in Fig. 3b. The abundance maps of these three elementary spectra across the FOV are presented in Fig. 3c. These elementary spectra correspond to three distinct spectral signatures: one representing the specimen matrix, another representing the epidermal regions of the leaf (referred to as Leaf Exterior), and the third representing the internal tissues (referred to as Leaf Interior) situated between the upper and lower epidermal layers.

The difference in fluorescence between the fossil leaf interior and exterior is consistent with our expectations. In living plants, the interior and exterior of a leaf will fluoresce differently^33^. In addition, TEM analyses of fossil leaves from the Miocene Clarkia site indicate that the interior and exterior of the fossil leaves preserve differently, which might also influence their fluorescence^34^.

The choice of elementary spectra for spectral unmixing involved a comprehensive approach, combining qualitative and quantitative assessments to select the appropriate elementary spectra. For instance, in Fig. 2, two elementary spectra were initially chosen to address the spectral complexity of the sample. Addition of a third spectrum resulted in a misleading attribution of leaf signal to the adjacent matrix in unmixing, which we knew not to be the case. In contrast, for Fig. 3, three elementary spectra were necessary due to distinct differences between the leaf’s interior and exterior portions. When we applied three elementary spectra to the unmixing of scans taken on matrix material only, the amount of signal attributed to the third unmixing component was low (i.e., within the noise level).

The three elementary spectra used exhibited considerable spectral overlap, which necessitated the use of a constraint on the abundance values when applying the spectral unmixing procedure. Unconstrained unmixing allows for both positive and negative abundance values and provides an analytical solution for the abundance values (Eq. 3 in Materials and Methods), which speeds up processing of data. However, in certain scenarios, allowing for negative abundance values can be disadvantageous, as it may allow for too much freedom in choosing the optimal combination of the three abundances, which is equivalent to noise in the spatial distribution maps of intensities obtained after unmixing.

To ensure accurate material abundance estimates, we imposed a nonnegativity constraint on the abundance values using an iterative least squares unmixing procedure. In order to enforce a nonnegativity constraint on the abundance values, we solved Eq. (2) for the abundance values iteratively using an open-source Matlab code^35^. The abundance maps obtained from spectral unmixing for the FOV shown in Fig. 3 exhibited substantial improvements compared to the unconstrained approach; the unconstrained unmixing with multiple overlapping elementary spectra of the same FOV led to a severe escalation in the pixel-to-pixel noise (See Supplementary Fig. 1) which overwhelmed the ability to distinguish certain features in the abundance maps. The constrained approach provided clearer and more reliable spatial mappings, enabling better visualization and analysis of distinct spectral components in the leaf compression fossil.

### Amber

As fossilized tree resin, amber is itself a type of plant fossil, and it also commonly embeds exceptionally well-preserved fossils of other plant parts such as leaves, twigs, and flowers^10,36^. Chemical characterization and classification of amber, and specifically tracing isolated amber pieces back to the tree that produced them, is a common research theme^37–40^. Fossil inclusions in amber typically exhibit exquisite external morphological preservation, although the preservation of internal morphology and the original tissue composition varies widely^41–43^. Fluorescence originating from the cuticle of a twig preserved in Baltic amber has been reported, with a similar green color as in the fluorescence of extant plant cuticle, although this analysis was carried out destructively, because the specimen was cut open to expose the twig for fluorescence imaging^44^. Fossils in amber are considered to be a very likely source for original biomolecule preservation.

To characterize the fluorescence emitted by fossil inclusions, we performed microspectroscopic scans of various Baltic Amber samples containing such inclusions. The scanned regions encompassed areas with fossil inclusions as well as regions consisting solely of amber resin. The Baltic Amber samples had an average thickness of approximately 10 mm, providing complete encapsulation of the inclusions, which themselves were a few millimeters thick. The PSF along the optical axis of the two-photon imaging system used in this study has a full width at half maximum (FWHM) of 1.6 µm. This means that only a thin layer around the focal point is excited, and the spectral data acquired represent fluorescence emission from this specific focal plane. Consequently, our imaging system effectively measured a few millimeters deep, allowing us to capture spectral information from the inclusions.

By virtue of the increased thickness of the amber samples, the potential for laser-induced damage was substantially reduced, compared to the more fragile leaf compression fossils. Leveraging this advantage, we slightly increased the power-per-excitation voxel to achieve a major reduction in the exposure time. By delivering an average power of 0.5 mW per pixel and an exposure time of 7 ms per pixel, we efficiently captured spectrally resolved fluorescence signals from a 1 mm² cross-sectional area within approximately 5.5 seconds. This comparatively short exposure time allowed us to perform multiple scans of each FOV, employing a range of excitation wavelengths. Specifically, we scanned each FOV six times, starting at an excitation wavelength of 760 nm and increasing by increments of 40 nm for each subsequent scan. Each excitation wavelength produced a microspectroscopic stack of images with 40 different wavelength channels, each having a bandwidth of approximately 5 nm. Although we analyzed only Baltic amber in this study, we expect the method would work equally well on any type of amber.

**Fig. 4:**
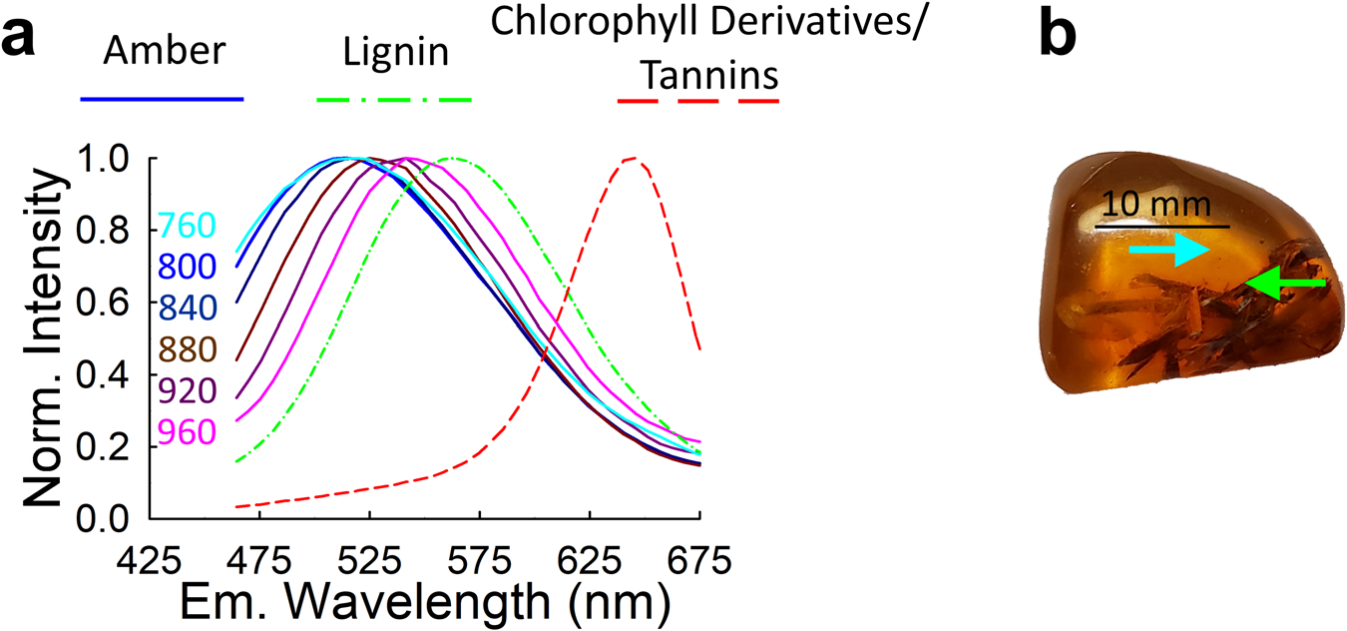
Illustration of the variability in emission spectra among regions within a sample for different two-photon excitation wavelengths for FMNH PE 93421, a specimen of Eocene Baltic Amber containing plant debris. **a** The solid lines are different emission spectra of amber obtained from the plant-free regions pointed at by the cyan arrow in the photograph shown in (**b**) and correspond to the excitation wavelengths indicated on the left side of each solid line. The dashed lines represent unique spectral signatures (labeled Lignin and Chlorophyll Derivatives/Tannins) obtained by scanning the region containing fossil inclusions indicated by the green arrow in (**b**) using 760 nm excitation. The excitation wavelength of 760 nm was chosen for all measurements on fossil inclusions to maximize the spectral (or wavelength) separation between the emission spectrum of amber and that of Lignin.

Figure 4a displays examples of emission spectra obtained from various regions within a single Baltic Amber sample containing fossil inclusions (which is pictured in Fig. 4b). It is noteworthy that the emission spectrum of Baltic Amber itself is influenced by the excitation wavelength employed during the measurement. Baltic amber is extremely chemically complex and includes many different components, some of which likely contribute to fluorescence^45,46^. The variation in fluorescence emission of Baltic Amber at different excitation wavelengths is likely due to different excitation efficiencies of the various fluorescent molecules in the amber. The solid lines in the plot of Fig. 4a show the average emission spectrum of Baltic Amber collected at various excitation wavelengths; the color of each line indicates the particular excitation wavelength the emission spectrum was collected at, following the color code given on the left of the plot. Notably, as the excitation wavelength increases, the peak of the Baltic amber emission spectrum shifts towards longer wavelengths, and the spectrum becomes narrower.

The excitation-dependent spectral properties of Baltic Amber enabled us to carefully choose an excitation wavelength to ensure maximum separation in peak emission wavelength between the emission of the amber and the various fluorescent species of the fossils embedded within the resin. Based on the data shown in Fig. 4a, in our further analysis, we chose an excitation wavelength of 760 nm, which facilitated accurate characterization and analysis of the unique spectral features exhibited by the amber and the plant inclusions.

As we shifted our focus to the fluorescence of the fossil inclusions themselves, it became evident that their emission spectra exhibited variability across different locations within the samples. To facilitate analysis, we categorized the inclusion emission spectra into two distinct groups based on their shape and peak wavelength. The detailed procedure used to determine these elementary spectra is described in the subsection titled “Determination of elementary spectra” in the Materials and Methods section.

The elementary emission spectrum with a peak at about 560 nm (dash-dotted line of Fig. 4a) falls within the range (~540–580 nm) attributed to lignin, cellulose or the cellulose–lignin complex in plant cell walls^14^. This spectrum was only observed in the two samples of Baltic Amber that contain debris with the appearance of stems or dry leaf matter, and not in the specimen of Baltic Amber containing a fragment of conifer leaf. Stems in particular would be more heavily lignified than leaves, potentially explaining this difference. Alternatively, analyses of fossil animal inclusions in amber have demonstrated a wide range of preservational variability, ranging from most internal tissues preserved to no internal tissues preserved^41,43^. The preservation of plants in amber has been studied less^41^, but if similar patterns hold true, the lack of lignin fluorescence in the leaf in Baltic Amber may simply represent a lack of preserved lignified tissues in this specimen. The elementary emission spectrum with a peak at ~650 nm (dashed red line of Fig. 4a), may be attributable to chlorophyll derivatives^14^, or to tannins^3^, which would be expected to occur in both stems and leaves.

**Fig. 5:**
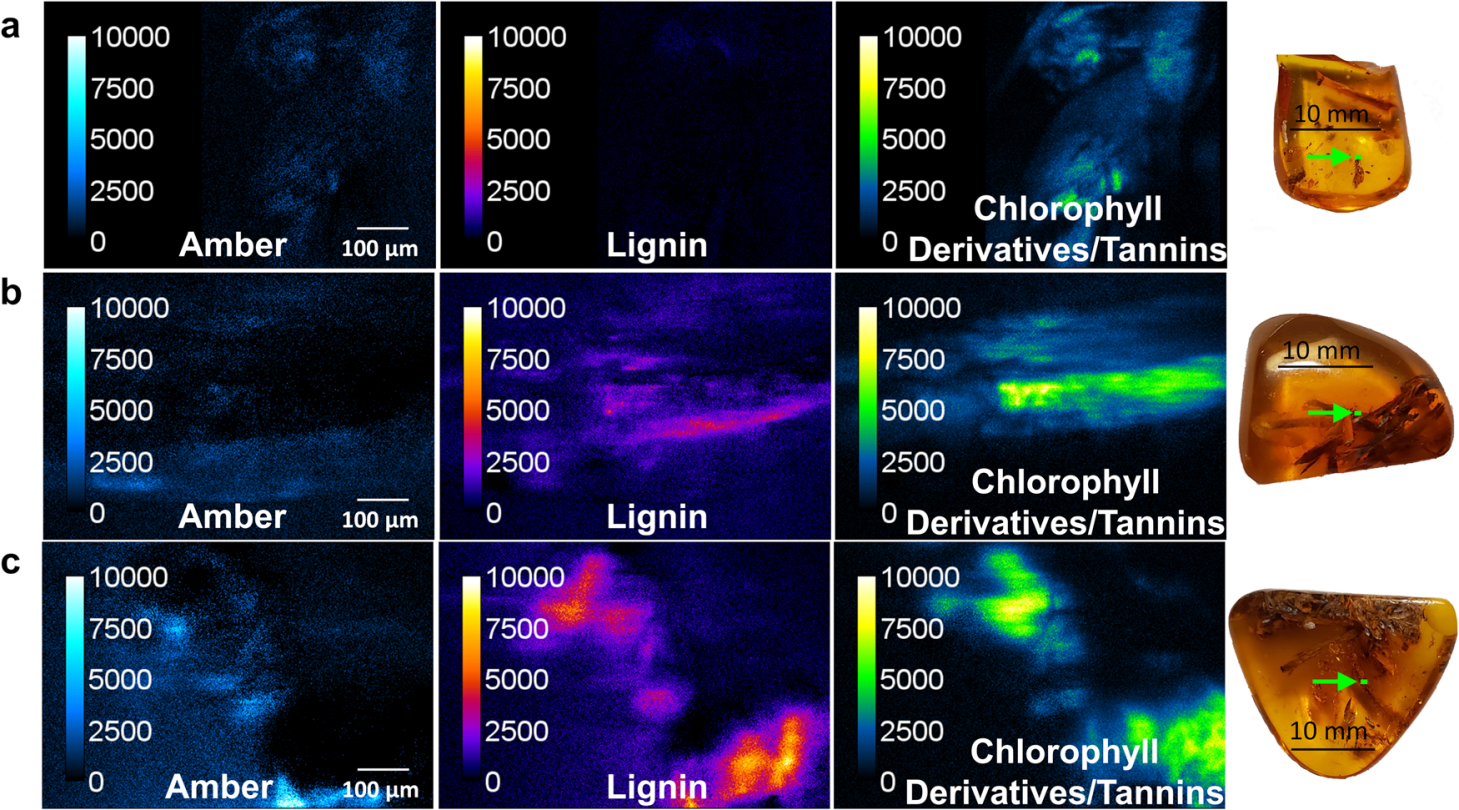
Abundance maps on the left obtained from spectral unmixing of microspectroscopic image stacks acquired using two-photon excitation of the areas indicated by the green arrows in the images on the right for the three specimens analyzed of Eocene Baltic Amber with plant inclusions. The excitation wavelength was 760 nm for all samples. Spectral unmixing was performed by using Eq. 2 in the text and three distinct elementary spectra: Amber, Lignin, and Chlorophyll Derivatives/Tannins. The array of abundance maps demonstrates the ability of the spectral unmixing procedure to accurately quantify the abundance of Lignin and Chlorophyll Derivatives/Tannins components independently in different scenarios: (**a**) when only Amber and Chlorophyll Derivatives/Tannins signals overlap in the same region (FMNH PE 93422, Baltic Amber containing unidentified leaf), (**b**) when Amber and Lignin signals overlap in the same region (FMNH PE 93421, Baltic Amber containing plant debris), and (**c**) even when the signals from Lignin and Chlorophyll Derivatives/Tannins overlap spatially (FMNH PE 93423, Baltic Amber containing plant debris).

**Fig. 6:**
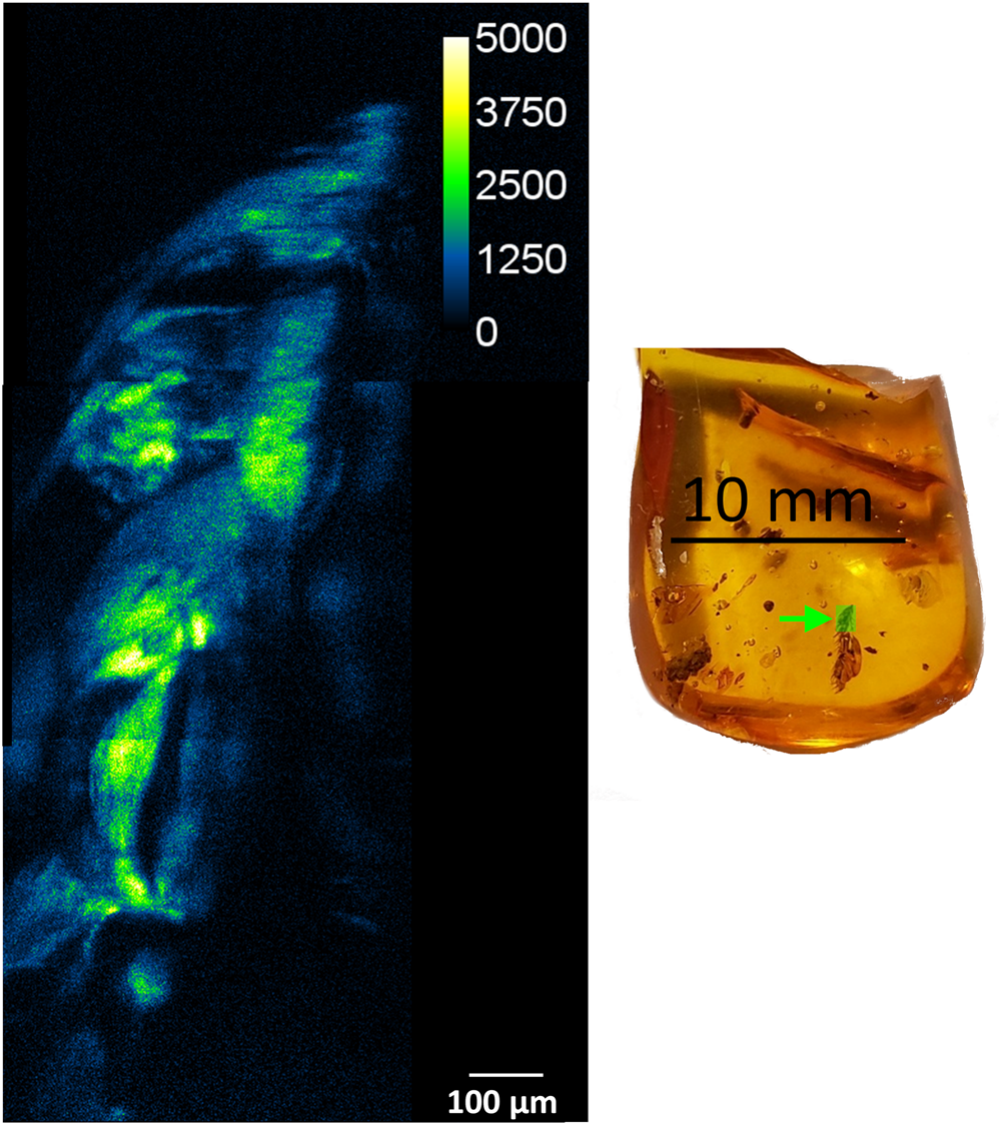
Abundance map obtained from spectral unmixing of microspectroscopic stacks obtained from two-photon excitation of three adjacent regions within a single specimen of Eocene Baltic Amber containing an unidentified leaf (FMNH PE 93422). The green rectangular region in the photograph on the right was illuminated by the ultrafast laser at a wavelength of 760 nm. The map shows the well-defined spatial distribution of the fluorescent species comprising the Chlorophyll Derivatives/Tannins fluorescent species, including the boundary between the inclusion and the surrounding amber. Variations in the distribution of this fluorescent species most likely represent its variable preservation throughout the specimen.

The outcome of the spectral unmixing analysis conducted on the microspectroscopic stacks obtained from three different Baltic Amber samples using an excitation wavelength of 760 nm is illustrated in Fig. 5. The figure presents abundance maps for each of the elementary spectra used in the unmixing procedure, namely Amber, Lignin, and Chlorophyll Derivatives/Tannins. The array of abundance maps in Fig. 5 provides a clear demonstration of the spectral unmixing procedure’s accuracy in independently quantifying the abundance of Lignin and Chlorophyll Derivatives/Tannins components across various scenarios. In Fig. 5a, we observe the effectiveness of spectral unmixing when Amber and Chlorophyll Derivatives/Tannins signals overlap within the same region of interest. In Fig. 5b, we observe distinct regions exhibiting different fluorescence characteristics. One region shows contributions of both Amber and Lignin, while the neighboring region includes primarily contributions from fluorescing species associated with Lignin. The spectral unmixing procedure plays a crucial role in accurately demarcating a clear boundary between these adjacent regions, allowing for the precise separation of the fluorophores representing Lignin from the region composed of a mixture of fluorophores representing both Amber and Chlorophyll Derivatives/Tannins. Finally, the abundance maps of Fig. 5c demonstrate the key ability to separately quantify the signals from Lignin and Chlorophyll Derivatives/Tannins when the two overlap in space. The abundance maps stitched together of the fossil material in three adjacent regions within the Baltic Amber sample (Chlorophyll Derivatives/Tannins) are pictured in Fig. 6. The map shows the well-defined spatial distribution of the materials identified by Chlorophyll Derivatives/Tannins, including the boundary between the inclusion and the surrounding amber.

Fossilization patterns are well-studied for arthropod inclusions in amber and are known to be highly variable, including within individual bio-inclusions [41]. Fossilization of plants in amber is probably similarly variable and likely to also result in inconsistent preservation of original material across an inclusion. Therefore, spatial variations in the Chlorophyll Derivatives/Tannins fluorescence emission spectrum can be attributed to some regions of the plant fossil exhibiting good preservation of this fluorescent species, and other regions exhibiting poor preservation.

Taken together, the results shown in Figs. 5, 6 highlight the method’s potential to provide valuable insights into the composition and properties of amber-embedded fossils. This approach not only accurately identifies boundaries between regions containing different fluorophores, but it also provides quantitative measurements of their abundance, even when they are in the proximity of each other.

### Silicified wood

Silicified wood is one of the most common and best-preserved types of fossil wood^47^. It often includes details of both cellular and subcellular structures^48^. However, much of the research into the composition and chemistry of silicified wood has focused on the inorganic component, specifically the structure and chemical composition of the silica^47,49^. Organic matter in the cell walls, for example, is preserved but it has not been sufficiently characterized^20^. This is largely due to the difficulty of in situ chemical analyses on the cellular scale of tiny amounts of organic material surrounded by large amounts of mineral. By improving our knowledge of the intricate interactions between organic and inorganic processes in the fossilization of plant material, we can gain a better insight into the evolution of ancient ecosystems and the environmental conditions that shaped them.

We applied two-photon microspectroscopy to various specimens of silicified wood to better gauge our ability to determine information regarding the organic material present in these samples. Fluorescence images were acquired from petrographic thin sections mounted on glass slides (see *Materials* section). By using such thin sections, we were able to use a high numerical aperture (NA) 60x oil immersion objective (infinity corrected plan Apo, NA = 1.4, WD = 0.13 mm; Nikon Instruments Inc.) for imaging, resulting in enhanced resolution.

**Fig. 7:**
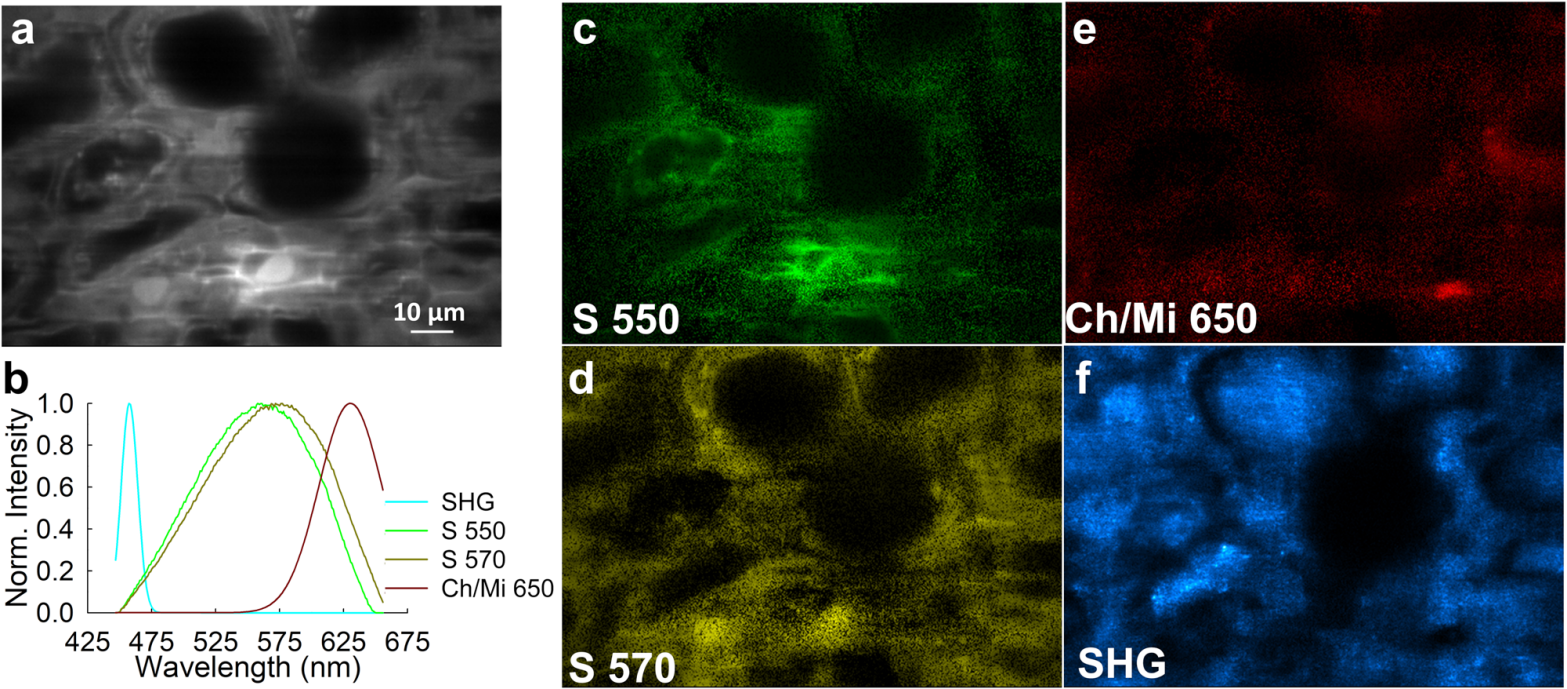
Typical microspectroscopic results obtained from two-photon excitation of a thin section of CBW-021, silicified angiosperm wood from the middle Eocene Casa Blanca Formation in Laredo, Texas, USA. **a** Spatial distribution map generated by averaging the fluorescence signals obtained over 200 wavelength channels ranging from 440 nm to 640 nm (shown for enhanced visualization of the cellular detail). **b** Plots of the elementary spectra used in the spectral unmixing procedure defined by Eq. 2 in the text. Two of the spectra contain major contributions from silica minerals, one with a peak near 550 nm (S 550) and one with a peak near 570 nm (S 570). These two spectra potentially contain small contributions from varying amounts of residual organic material left in the plant cell wall (lignin, cellulose, cellulose–lignin complex) which would explain the slight shift in the emission peak wavelength. A third elementary spectrum with an emission peak near 650 nm (Ch/Mi 650) potentially corresponds to chlorophyll derivatives, tannins, or trace minerals within the sample. Finally, a fourth elementary spectrum represents a second harmonic generated signal (SHG)^55–57^. **c**–**f** Abundance maps obtained by spectral unmixing of the microspectroscopic image stacks of silicified wood using elementary spectra plotted in **b**. The silica and Ch/Mi 650 abundance maps (**c**–**e**) were collected using an excitation wavelength of 760 nm, while the SHG abundance map (**f**) was collected using an excitation wavelength of 920 nm. For the data obtained by two-photon excitation at 760 nm, all the values in the SHG elementary spectrum were set to zero, as no SHG signal was detected at this excitation wavelength.

Figure 7 illustrates the results of spectral unmixing analysis applied to the microspectroscopic stacks obtained from a thin section of silicified wood with preserved cellular structure (Fig. 7a). To perform the unmixing process on data obtained at an excitation wavelength of 760 nm, we employed three elementary spectra, each of which are plotted in Fig. 7b. Among these spectra, two displayed a complex and broad composition, suggesting the presence of multiple fluorescing entities. The distinction between the two spectra was characterized by a subtle shift in their emission peaks, with one peaking near 550 nm (referred to as S 550, Fig. 7c), and the other near 570 nm (referred to as S 570, Fig. 7d). Silica minerals, which coat the remnant organic matter of the cell walls or fill the inter- and intra-cellular spaces of the wood, undoubtedly contribute to both S 550 and S 570. For example, laser-induced fluorescence of quartz from the La Sassa site in Italy produces a fluorescence emission spectrum with a peak at 547 nm, due to various impurities and activators^50^. In particular, as quartz develops from amorphous silica, self-trapped excitons can form^51,52^ that can produce this specific fluorescence emission spectrum^50^. Silicification of wood most commonly occurs as a continuous process in which a crystalline end product (quartz) is produced over a long period of time from an initial amorphous silica precipitate^47^; self-trapped excitons may have developed during this process. However, considering the broadness of the two spectra as well as the slight shift in peak wavelength between them, it is also conceivable that other components such as lignin, cellulose, or the cellulose–lignin complex present in plant cell walls^14^ contribute to the observed fluorescence. This is consistent with a previous Raman spectroscopy study of mineralized wood which demonstrated that lignin and, to some extent, cellulose do preserve in mineralized wood^53^. The third spectrum (Ch/Mi 650, Fig. 7e), with a peak at ~650 nm is within the range (600–750 nm) usually attributed to chlorophyll or its derivatives^14^. Chlorophyll is not typically expected to be found in wood, although red fluorescence due to chlorophyll has been noted in extant poplar wood^54^. Tannins can also fluoresce with peak emission ranging from 500–650 nm, and are commonly found in wood^3^. This spectrum could also be attributed to trace minerals found in the sample, present in much lower concentrations than silica.

A second scan of the FOV shown in Fig. 7 was acquired using an additional excitation wavelength of 920 nm. This particular wavelength induced a phenomenon known as second harmonic generation (SHG), which results in the generation of a new signal at twice the frequency of the excitation light that is generated in certain crystallin structures^55–57^ and has also been detected in the wood of extant trees^58^. The microspectroscopic scans acquired at 920 nm were unmixed using four elementary spectra: S 550, S 570, Ch/Mi 650, and a narrow emission spectrum with a peak wavelength of 460 nm corresponding to the SHG signal. All four of these elementary spectra are plotted in Fig. 7b, and the abundance map of the SHG signal is shown in Fig. 7f.

The SHG signal can be attributed to multiple potential sources. Tai et al.^58^ utilized spectrally resolved two-photon microscopy to examine the fluorescence emission from various wooden musical instruments, including both modern and centuries-old instruments made of spruce. Their spectrally resolved fluorescence images revealed distinct subcellular structures within the spruce samples, including the middle lamella, tracheid cell wall, and bordered pits. Notably, large SHG signals were observed in both the modern and aged spruce samples, attributed to the cellulose crystal structures. This phenomenon was particularly pronounced in the border pit membranes and tracheid cell walls. However, it remains to be determined whether the signal observed in the silicified wood samples in our study originates primarily from cellulose. The resilience of cellulose in fossils is relatively limited compared to that of lignin^59^. Therefore, a second explanation to the SHG signal could be crystals formed by the silica minerals filling the spaces within and between cells. Future investigations will explore further whether the organic material that remains can be detected via fluorescence.

## Discussion

We have shown that spectrally resolved fluorescence micrographs obtained from two-photon microspectroscopy imaging of fossil plants embedded in various types of matrices can be used in conjunction with spectral unmixing to obtain separate images of the matrix and the original biological material or chemical products generated during fossilization. The broadness of the fluorescence spectra observed in the samples measured in this study emphasizes the crucial necessity for high spectral resolution from our microspectroscope. This resolution is crucial as we aim to distinguish subtle variations in emission spectra, which can originate from residual fluorescence species that retain the characteristics of the original biological system. Depending on the composition of the fossil material, spectral unmixing was performed with two or three spectral components. The latter required additional mathematical constraints to reduce noise and increase the contrast of each of the unmixed spectral images.

Using the methods developed in our study, it is possible to apply two-photon microspectroscopy to a wide variety of fossil plants, including leaf compression fossils, fossil inclusions in amber, and silicified wood, to quantify their fluorescence spectra, which is the first step to interpreting and understanding the source of the fluorescence known to be widespread in fossil plants^9^. Our initial preliminary interpretations of the emission spectra presented herein suggest that some are consistent with the expected fluorescence emission spectra of original fluorescent tissues, primarily to the resistant tissues of the fossils (lignin/cellulose) and possibly to chlorophyll derivatives or tannins, suggesting that fossil plants can retain evidence of their original biological fluorescence. Therefore, investigations into fluorescent fossil plants using the protocols developed in this study may reveal information about the under-studied and as yet poorly understood role of plant fluorescence in ancient ecosystems. However, minerals or materials produced during fossilization may also contribute to the measured fluorescence emission spectra, and so additional chemical analyses to fully characterize the organic composition of fluorescent fossil plants are needed to ground-truth these observations and assist in interpreting the fluorescence emission spectra more precisely.

Understanding the composition of fossils, particularly which original tissues and biomolecules preserve, as well as fossilization itself are major areas of paleontology research^60^. Most techniques for analyzing the organic composition of fossils, such as GC-MS, are destructive, which means the fossil material used for analysis is not accessible for further research, and that the spatial distribution of different tissues and biomolecules cannot be determined. For this reason, there is a current major effort to develop non-destructive methods to analyze fossils^61,62^. This is particularly relevant for the biomolecule analysis of fossil inclusions in amber. A major hurdle for biomolecule analysis in fossil materials in general is modern contamination; however, the amber surrounding a fossil inclusion prevents modern contamination. By applying the methods that we developed for the fluorescence spectroscopy of inclusions in amber through the protective outer layer of amber, fluorescent biomolecules could be identified without exposing the inclusion to outside contamination. For example, some amino acids and peptides fluoresce^63^, and amino acids have been demonstrated (through destructive analyses) to preserve in a small percentage of fossils in amber^64^. The methods that we have developed here could be used in combination with spectroscopy of fluorescent amino acid standards to determine non-destructively which fossils in amber preserve amino acids.

Although at this time we did not attempt to identify any fluorescence spectra indicative of the preservation of original biological fluorophores used for visual signaling to animals, if such fluorophores are preserved in any fossils, they could be quantified in a non-destructive way using these methods.

While the methods we developed are directly relevant to the study of fossil plant fluorescence, they are more broadly applicable. Fossils of other organisms, such as animals, fungi, and microfossils, can preserve following similar fossilization pathways as plants, including as organic compressions^65^, as inclusions in amber^10^, and through silicification^66^. Some non-plant fossils are also known to fluoresce^67,68^. Moreover, applying two-photon fluorescence microspectroscopy to modern biological samples has some of the same challenges as carrying out such analyses on fossil plants; for example, modern tissues often contain a mixture of fluorophores, fluorescence emission can vary on a cellular or subcellular scale, and organic material is susceptible to photodamage^3,69^. Therefore, the methods developed in this study would likely be a valuable tool for analyzing fluorescent fossils of animals, fungi, or microorganisms as well as fluorescent biological specimens.

Our data analysis reveals that for some samples, choosing a different excitation wavelength leads to different emission spectra, possibly because different fluorescent species are excited. A very enticing opportunity for extracting additional insights lies in the integration of the excitation spectrum into the analysis of microspectroscopic datasets acquired from amber samples with fossil inclusions. By using the excitation wavelength as a fourth dimension alongside spatial coordinates and emission wavelength, we can uncover a wealth of valuable information on the composition and properties of these fascinating fossils. Supplementary Fig. 2 illustrates an example where this added dimension can potentially reveal variations in the chemical composition of regions that have similar emission spectrum characteristics; two different regions, possessing similar emission spectrum (Lignin) exhibit largely different excitation spectra (Lignin’ vs Lignin”). This four-dimensional data stack (with two spatial and two spectral dimensions) will be incorporated into a future study that will focus on the details of plant inclusions in various types of amber, allowing for the differentiation among fluorophores exhibiting the same emission elementary spectrum.

It is also possible, of course, to incorporate three spatial dimensions by successively scanning the sample at different depths. This is routinely done using two-photon microscopes (including the one used in this study), though its use in conjunction with additional spectral dimensions will require the development of powerful visualization studies.

## Materials and Methods

### Materials

Three sets of fossil plants that had undergone different fossilization processes were selected for analysis. The first set consisted of fossil plant compressions, the second was comprised of plant inclusions in ancient amber, and the third was a piece of silicified wood.

Two specimens of fossil plant compressions from the Miocene (~16 Ma) Clarkia deposit in Idaho, USA were collected by VEM and CTG in August 2021, with permission from the land owner of the site. Both specimens are accessioned in the Milwaukee Public Museum (MPM). Both specimens, a leafy twig of *Taxodium* sp. (MPM Pb2853) and an unidentified dicot leaf (MPM Pb2854) were collected from site P-33, which is locally called the Fossil Bowl or Fossil Racetrack locality^70^.

Three specimens of plant inclusions in Eocene (~45 Ma) Baltic Amber were obtained from the Invertebrate Paleontology collection of Field Museum of Natural History (FMNH) in Chicago, USA, for this study. Two of the specimens, FMNH PE 93421 and FMNH PE 93423, contain plant debris consisting of dry stems or leaf material. One specimen, FMNH PE 93422 contains a fragment of a conifer leaf.

One specimen of silicified angiosperm wood was from the middle Eocene Casa Blanca Formation in the spillway of Laredo, Texas, USA. This specimen is accessioned into the fossil wood inventory system at the University of Bonn as CBW-021. A standard, uncovered petrographic thin section of CBW-021 was prepared by the National Petrographic Service.

### Two-photon fluorescence microspectroscopy

Fluorescence images were acquired using a spectrally resolved two-photon optical microspectroscope^15^ consisting of a tunable femtosecond laser (MaiTai^TM^, Spectra Physics, Santa Clara, CA), an inverted microscope (Nikon Eclipse Ti^TM^, Nikon Instruments Inc., Melville, NY) equipped with an infinity-corrected, plan fluorite objective lens (10×, NA = 0.3, WD = 16 mm; Nikon Instruments Inc.), and an OptiMiS scanning/detection head (Aurora Spectral Technologies, Grafton, WI). The majority of laser scanning fluorescence microscopes utilize a serial point scanning approach, whereby only a single excitation voxel is being sampled at any given time, which means that only a small fraction of the total scan time is devoted to any given pixel. Utilizing a true line-scanning protocol allows for hundreds of voxels to be excited simultaneously. The incorporation of a line-shaped excitation beam increases the sensitivity of the device by two orders of magnitude compared with serial point scanning devices. The samples were scanned using a line-shaped excitation beam with a power of 0.2-0.5 mW/voxel and an integration time of 7–35 ms per pixel. The integration time and excitation power varied depending on the specific sample measured. Please refer to the Results section for detailed information on the excitation parameters corresponding to each sample type.

The line-shaped beam was swept over a field of view corresponding to an area in the sample of 0.7 mm × 0.48 mm. Initially, all fields of view were scanned using an excitation wavelength of 760 nm. For certain types of samples (See Results section for more details), additional scans were conducted at varying excitation wavelengths—from 760 nm to 960 nm, with 40 nm increments—for each field of view. Each excitation scan produced a collection of microspectroscopic images, typically consisting of 200 wavelength channels with a bandwidth of approximately 1 nm. The image size for each emission wavelength channel was 440 × 300 pixels. To achieve superb spectral resolution, adjustable down to 1 nm, these optical microspectroscopes utilize a combination of a diffraction grating and an electron multiplying CCD (EMCCD) camera (iXon X3 897, Andor Technologies, Belfast, UK). This setup allows each row on the EMCCD to function as a separate wavelength channel. For the few samples that exhibited major variations in emission spectra between fluorescing components, the wavelength channel bandwidth was increased to 5 nm. This adjustment resulted in a reduced total number of spectral channels (40), but it improved the fluorescence signal-to-readout noise ratio of the detector. The lateral resolution of the instrument was measured to be 500 nm^15^.

### Linear mixture model for spectral unmixing

The emission spectrum at each pixel in the microspectroscopic images was deconvoluted using a linear mixture model (LMM). In an LMM, the measured emission spectrum for a particular pixel, ${{{{{\boldsymbol{S}}}}}}$, is modeled as a linear combination of *l* elementary spectra multiplied by a coefficient proportional to the abundance of the corresponding material, ${k}_{i}$. The *l* elementary spectra (also referred to as components or endmembers) represent the purest spectra of the individual materials that make up a region in the sample. Elementary spectra are assumed to be spectrally distinct and independent, meaning they do not depend on each other, and their combination in different proportions can account for the observed spectrum of any pixel in the image. If the normalized elementary spectra are denoted by vectors $({{{{{{\boldsymbol{s}}}}}}}_{{{{{{\boldsymbol{1}}}}}}},{{{{{{\boldsymbol{s}}}}}}}_{{{{{{\boldsymbol{2}}}}}}},\ldots ,{{{{{{\boldsymbol{s}}}}}}}_{{{{{{\boldsymbol{n}}}}}}})$, the measured intensity spectrum for a particular pixel may be written as:

1 $${{{\boldsymbol{\ S}}}}=\overline{\overline{{{\boldsymbol{\ s}}}}}\cdot {{{\boldsymbol{\ k}}}}+{{{\boldsymbol{\ n}}}}$$

where ${{{{{\boldsymbol{S}}}}}}$ is a vector of length *m* holding the measured intensity values across all *m* wavelength channels, $\overline{\overline{{{\boldsymbol{\ s}}}}}=[{{{\boldsymbol{\ s}}}}_{{{\boldsymbol{\ 1}}}}{{{\boldsymbol{\ s}}}}_{{{\boldsymbol{\ 2}}}}\ldots {{{\boldsymbol{\ s}}}}_{{{\boldsymbol{\ l}}}}]$ is an $m\times l$ matrix composed of the normalized emission spectra of the *l* individual endmembers, $${{{{{\boldsymbol{k}}}}}} \, {{{{{\boldsymbol{=}}}}}}\left[\begin{array}{c}{k}_{1}\\ \begin{array}{c}{k}_{2}\\ \begin{array}{c}\vdots \\ {k}_{l}\end{array}\end{array}\end{array}\right]$$ is a vector of the coefficients, or abundances, of each individual elementary spectrum (or endmember), and ${{{{{\boldsymbol{n}}}}}}$ is a vector of length *m* holding the experimental noise values for each wavelength channel.

The goal of the unmixing procedure is to extract the abundance vector, ${{{{{\boldsymbol{k}}}}}}$, which contains the amount of fluorescence emanating from each constituent comprising the mixture. This is accomplished by using a least squares minimization procedure, given by:

2 $${\min }_{{{\boldsymbol{\ k}}}}||{{{\boldsymbol{\ S}}}}-\overline{\overline{{{\boldsymbol{\ s}}}}}\cdot {{{\boldsymbol{\ k}}}}|{|}_{2}^{2}$$

Equation (2) represents the minimum value of the second norm square of the expression within ||∙||. Taking the derivative of the expression to be minimized by ***k*** as zero, and rearranging the equation yields an analytical solution to the abundance vector ${{{{{\boldsymbol{k}}}}}}$, as

3 $${{{\boldsymbol{\ k}}}}={({\overline{\overline{{{\boldsymbol{\ s}}}}}}^{{{\boldsymbol{\ T}}}}\cdot \overline{\overline{{{\boldsymbol{\ s}}}}})}^{{{\mathbf{-1}}}}\cdot {\overline{\overline{{{\boldsymbol{\ s}}}}}}^{{{\boldsymbol{\ T}}}}\cdot {{{\boldsymbol{\ S}}}}$$

where the superscript ^***T***^ indicates the transposed matrix. Equation (3) does not enforce any nonnegativity constraint on the components of the abundance vector ${{{{{\boldsymbol{k}}}}}}$ and is therefore commonly referred to as the unconstrained least squares estimate (UCLS) of the abundance.

To enforce a nonnegativity constraint on the abundance values, we solved Eq. (2) iteratively using the open-source Matlab code lsqnonnegvect.m, which implements a vectorized version of the Matlab lsqnonneg function^35^. The vectorized approach speeds up the process of solving multiple non-negative least square fits of independent data vectors, facilitating efficient pixel-level spectral unmixing with a non-negativity constraint on the abundances applied.

### Determination of elementary spectra for spectral unmixing

The elementary fluorescence spectra $({{{{{{\boldsymbol{s}}}}}}}_{{{{{{\boldsymbol{1}}}}}}},{{{{{{\boldsymbol{s}}}}}}}_{{{{{{\boldsymbol{2}}}}}}},\ldots ,{{{{{{\boldsymbol{s}}}}}}}_{{{{{{\boldsymbol{n}}}}}}})$, utilized for unmixing were obtained through a semi-qualitative procedure described here. One elementary spectrum (or ${{{{{{\boldsymbol{s}}}}}}}_{{{{{{\boldsymbol{1}}}}}}}$) was measured by imaging regions consisting only of substrate material, i.e., where the laser was situated in a region far from any leaf remains or other plant matter. For example, in leaf compressions, the matrix substrate surrounding the fossil is made up of various minerals and organic material. Similarly, for the amber samples with inclusions, the substrate is comprised exclusively of the amber resin. The average spectrum from these substrate regions was then taken as a single elementary spectrum (a single-column vector) in $\overline{\overline{{{{{{\boldsymbol{s}}}}}}}}$. Subsequently, additional elementary spectra were discovered by sampling numerous regions that contained visual evidence of embedded plant material. For each field of view acquired from these areas, 3 to 4 polygonal regions of interest (ROI) were manually delineated around distinct features in the spectrally resolved fluorescence images. The average emission spectrum was collected for each ROI. To separate the substrate spectrum from the average composite spectrum within these regions, unmixing was conducted by combining the substrate spectrum with one or more Gaussian functions, and the resultant sum provided the emission spectrum of that ROI with the substrate extracted. The extracted emission spectra from all samples were then grouped together based on their similarities in breadth and emission maximum. Finally, the average emission spectra within each finalized group were normalized to their maximum intensity value, resulting in a single elementary spectrum vector (${{{{{{\boldsymbol{s}}}}}}}_{{{{{{\boldsymbol{i}}}}}}}$), which served as a component in the spectral unmixing procedure.

### Implementation of the spectral unmixing procedure

By employing the aforementioned elementary spectra and fitting algorithm, we performed unmixing on the composite fluorescence spectrum obtained for each pixel in the microspectroscopic images of every field of view. The unmixing procedure with the ANC was utilized to generate all unmixed images, except for the panels depicted in Supplementary Fig. 1c that were generated using the unconstrained unmixing procedure. Both variants of the unmixing procedures were implemented using the data analysis software suite OptiMiS DC which was developed in-house and provides an intuitive GUI (https://sites.uwm.edu/raicu-research-group/software/). OptiMiS DC offers a wide range of tools for processing spectrally resolved fluorescence images, including algorithms designed to unmix each set of fluorescence images at the pixel level, thereby separating contributions from different fluorescent species into individual intensity maps.

### Statistics and Reproducibility

In this study, we gathered data on various fossil types, including silicified wood, leaf compression, and amber inclusions. For each fossil type, we collected data from at least five different specimens. Additionally, for each individual specimen, we captured images from a minimum of five different fields of view. The spectral deconvolution procedure was implemented using a least squares method. To generate elementary spectra from the substrate material to be used in the spectral deconvolution procedure, we sampled at least five distinct regions of interest from substrate material only, i.e., measurements obtained where the laser was situated in a region far from any leaf remains or other plant matter. From each of these regions, we calculated the average emission spectra by considering data from all pixels within those areas. Subsequently, we averaged these emission spectra at the region of interest (ROI) level to derive the final elementary spectrum. Detailed descriptions of all analysis methods can be found in the Methods section for a more comprehensive understanding.

### Reporting summary

Further information on research design is available in the Nature Portfolio Reporting Summary linked to this article.

### Supplementary information

Supplementary Information

Description of Additional Supplementary Files

Supplementary Data

Reporting Summary

## Data Availability

The datasets used in the current study are available at 10.6084/m9.figshare.24802719^71^. The numerical source data behind the graphs in the manuscript can be found in Supplementary Data. All specimens used in the study are available from their repositories, and can be identified by their specimen numbers: two specimens of leaf impressions are deposited in the Milwaukee Public Museum as specimen numbers MPM Pb2853 and MPM Pb2854; three specimens of plant inclusions in Baltic Amber are in the Field Museum of Natural History as specimen numbers FMNH PE 93421, FMNH PE 93423, and FMNH PE 93422; one specimen of silicified wood is in the fossil wood inventory system at the University of Bonn as specimen number CBW-021.

## Code availability

The custom software OptiMiS DC, developed in-house by our research group, is made available for the scientific community. Researchers interested in utilizing OptiMiS DC may access and download the software from the following website: https://sites.uwm.edu/raicu-research-group/software.
