# Supplementary material for: Two-photon excitation fluorescence microspectroscopy protocols for examining fluorophores in fossil plants
Source: Commun Biol. 2024 Jan 6;7:53. doi: 10.1038/s42003-024-05763-z (PMC10771488; doi:10.1038/s42003-024-05763-z)
Supplement: Supplementary file 1 — Supplementary Information [file 42003_2024_5763_MOESM1_ESM.pdf]

Supplementary Information

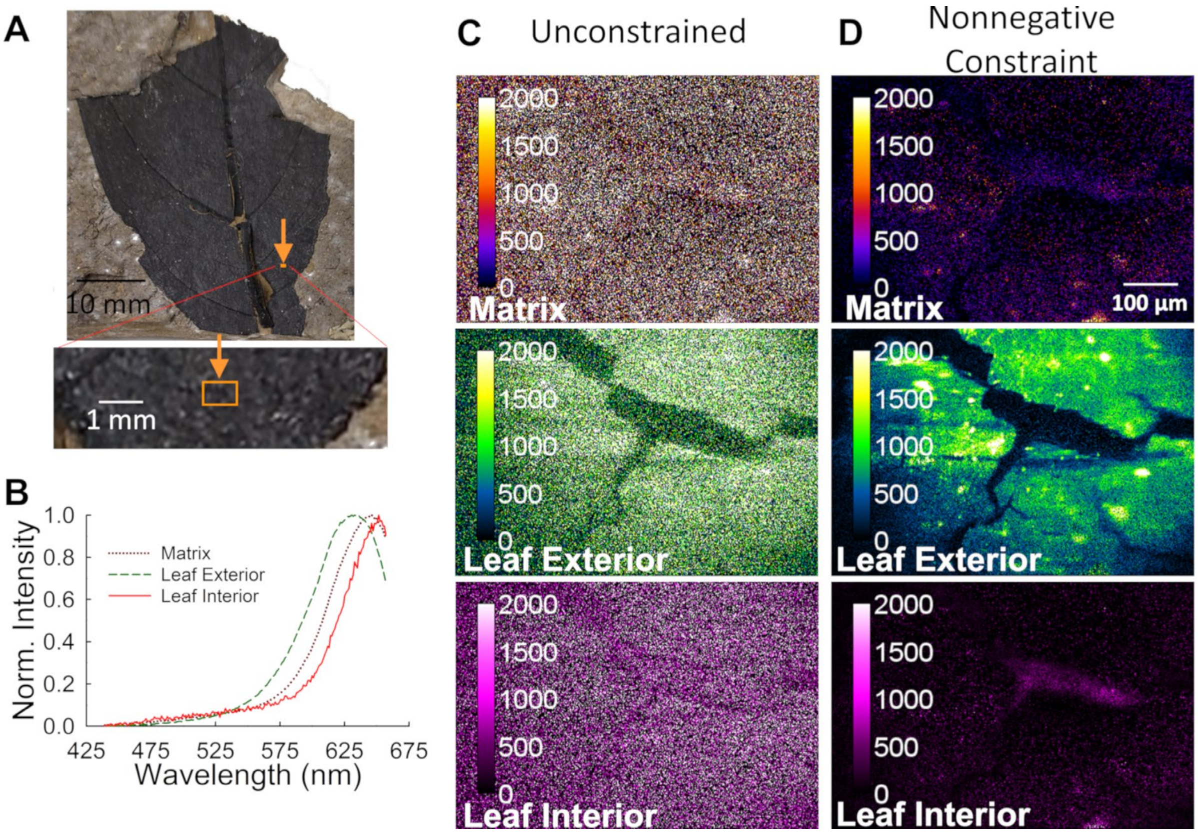

Supplementary Fig 1. Illustration of the necessity of using a nonnegative constraint on the pixel-level spectral unmixing when using three elementary spectra (A) Photo of a dicot leaf compression from the ~16 Ma Clarkia fossil deposit in Idaho, USA; the orange rectangle indicates the location of the laser beam on the fossil for the FOV displayed in C and D. The insert provides a magnified view of the scanned area. (B) Elementary spectra used for the unmixing procedure. For this particular field of view, three distinct spectral signatures were identified: one corresponding to the matrix, one representing the outer surface of the leaf, including possibly the waxy cuticle and the epidermis, (Leaf Exterior), and one representing the internal mesophyll (Leaf Interior) (C) Unmixing of the microspectroscopic images using an unconstrained least-squares

solution (UCLS) to the linear mixture model. In the UCLS case, the abundance vector k_i (see Eq. 1 in the main text) of each of the elementary spectrum of any of the components can be negative. When the three elementary spectra are spaced closely in peak wavelength, as is the case for the elementary spectra detected for this particular FOV, allowing for negative components permits for too much freedom in the fit and adds significant noise to the unmixing procedure. (D) Unmixing the same field of view (with the same elementary spectra) using an iterative unmixing with an abundance nonnegative constraint (ANC) imposed. The unmixing procedure was performed with a nonnegativity constraint on the abundance values (see the *Spectral Unmixing* subsection of the Materials and Methods) to ensure the accuracy of the results.

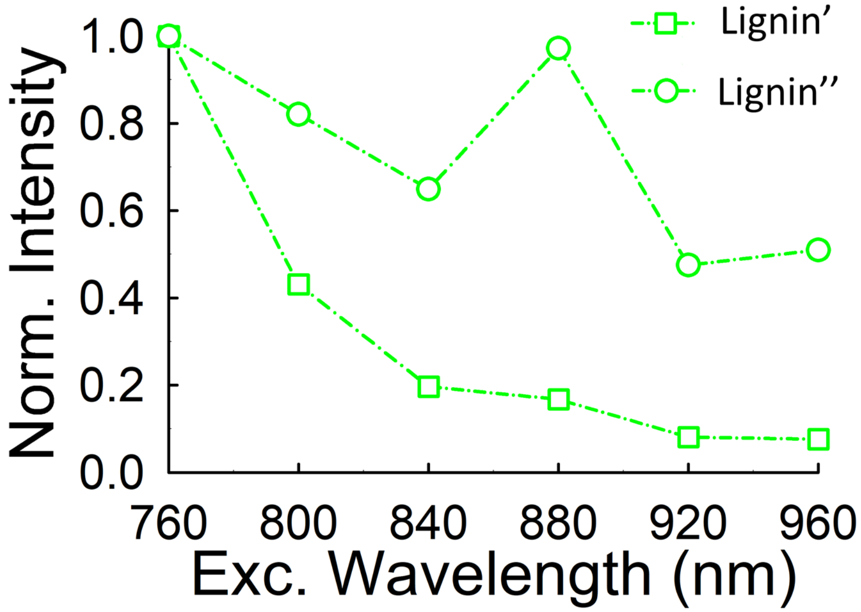

Supplementary Fig 2. Excitation spectra from two different ROIs within a sample of fossil inclusions embedded in amber. Abundance maps, similar to those shown in Fig 5 of the main text, were obtained from spectral unmixing of microspectroscopic image stacks acquired from Eocene Baltic Amber samples containing plant inclusions. The average intensity was then calculated from the pixels within manually drawn ROIs of the Lignin abundance maps (refer to Fig

4 in the main text) for six different excitation wavelengths. Plots of the average intensity vs excitation wavelength for two different ROIs are shown. This example demonstrates how the inclusion of excitation wavelength as an additional imaging parameter has the potential to reveal variations in the chemical composition of regions that exhibit similar emission spectrum characteristics. Notably, two distinct regions with comparable emission spectra (Lignin) displayed significantly different excitation spectra (Lignin’ vs Lignin’’).
