# Supplementary material for: Two-photon excitation fluorescence microspectroscopy protocols for examining fluorophores in fossil plants
Source: Commun Biol. 2024 Jan 6;7:53. doi: 10.1038/s42003-024-05763-z (PMC10771488; doi:10.1038/s42003-024-05763-z)
Supplement: Supplementary file 2 — Description of Additional Supplementary Files [file 42003_2024_5763_MOESM2_ESM.pdf]

Description of Additional Supplementary Files

File name: Supplementary Data
Description: The numerical data underlying each graph in the manuscript.
